# Expression of cyclins A and D and p21^(waf1/cip1)^ proteins in renal cell cancer and their relation to clinicopathological variables and patient survival

**DOI:** 10.1038/sj.bjc.6690634

**Published:** 1999-08

**Authors:** S Aaltomaa, P Lipponen, M Ala-Opas, M Eskelinen, K Syrjänen, V-M Kosma

**Affiliations:** Departments of Urology and Surgery and Pathology, Kuopio University Hospital, PO Box 1777, Kuopio, FIN-70210, Finland; Department of Pathology and Forensic Medicine, University of Kuopio, Kuopio, Finland

**Keywords:** cyclin A, cyclin D1, p21 ^(waf1/cip1)^, cell proliferation, renal cell carcinoma

## Abstract

We have studied 118 renal cell carcinomas to analyse the expressions of cyclins A and D1 and p21^(waf1/cip1)^, and their relationship to clinical and histopathological parameters as well as to clinical outcome. Cyclins A and D1 and cyclin-dependent kinase inhibitor p21^(waf1/cip1)^ were not expressed in normal renal tissue. Staining signals of cyclin D1 and p21^(waf1/cip1)^ were always nuclear but cyclin A was also expressed in the cytoplasm of the tumour cells. The mean (range) fractions of cyclin A, cyclin D1 and p21^(waf1/cip1)^-positive tumour cells were 2.2% (range 0–20%), 23.3% (range 0–90%) and 6.8% (range 0–70%) respectively. The expression of cyclin A was related to venous invasion, high nuclear grade, high mitotic rate, high Ki-67 and high PCNA expressions (*P* ≤ 0.006 for all). The expression of cyclin D1 was linked with age over 65 years, low nuclear grade and high p53 expression (*P* ≤ 0.05 for all). An inverse correlation was present between p21^(waf1/cip1)^ and cyclin D1 (*P* = 0.011). Cyclin A predicted survival in the entire study group (*P* = 0.0014), in T1–4/N0–2/M0 (*P* = 0.0007) and in T1–2/N0/M0 tumours (*P* = 0.0007). Cyclin A was also a powerful predictor of disease-free survival in T1–4/N0/M0 (*P* = 0.0027) tumours (*P* = 0.0007). Cyclin D1 and p21^(waf1/cip1)^ were not significantly related to survival or disease-free survival in any of the groups. In the entire material the independent prognostic factors were the presence of distant metastases (relative risk (RR) 5.16, *P* < 0.001), T category (RR 2.68, *P* < 0.001), Ki-67 expression (RR 1.02, *P* = 0.026) and cyclin A expression (RR 1.12, *P* = 0.001). The independent predictors in T1–4/N0/M0 tumours were T-category (RR 2.67, *P* = 0.001) and cyclin A (RR 1.21, *P* < 0.001), and in T1–2/N0/M0 tumours the only significant predictor was cyclin A (RR 1.19, *P* = 0.0002). In renal cell carcinoma, cyclin A is a powerful and independent prognostic factor in all clinical stages of the disease, whereas cyclin D1 and p21^(waf1/cip1)^ have no prognostic value. © 1999 Cancer Research Campaign


*British Journal of Cancer* (1999) **80**(12), 2001–2007
© 1999 Cancer Research Campaign
Article no. bjoc.1999.0634

### Expression of cyclins A and D and p21$^{(waf1/cip1)}$ proteins in renal cell cancer and their relation to clinicopathological variables and patient survival

**S Aaltomaa[1], P Lipponen[3], M Ala-Opas[1], M Eskelinen[2], K Syrjänen[3,4] and V-M Kosma[3,4]**

Departments of [1]Urology and [2]Surgery and [3]Pathology, Kuopio University Hospital, PO Box 1777, FIN-70210 Kuopio, Finland; [4]Department of Pathology and Forensic Medicine, University of Kuopio, Kuopio, Finland

**Summary** We have studied 118 renal cell carcinomas to analyse the expressions of cyclins A and D1 and p21$^{(waf1/cip1)}$, and their relationship to clinical and histopathological parameters as well as to clinical outcome. Cyclins A and D1 and cyclin-dependent kinase inhibitor p21$^{(waf1/cip1)}$ were not expressed in normal renal tissue. Staining signals of cyclin D1 and p21$^{(waf1/cip1)}$ were always nuclear but cyclin A was also expressed in the cytoplasm of the tumour cells. The mean (range) fractions of cyclin A, cyclin D1 and p21$^{(waf1/cip1)}$-positive tumour cells were 2.2% (range 0–20%), 23.3% (range 0–90%) and 6.8% (range 0–70%) respectively. The expression of cyclin A was related to venous invasion, high nuclear grade, high mitotic rate, high Ki-67 and high PCNA expressions ($P \leq 0.006$ for all). The expression of cyclin D1 was linked with age over 65 years, low nuclear grade and high p53 expression ($P \leq 0.05$ for all). An inverse correlation was present between p21$^{(waf1/cip1)}$ and cyclin D1 ($P = 0.011$). Cyclin A predicted survival in the entire study group ($P = 0.0014$), in T1–4/N0–2/M0 ($P = 0.0007$) and in T1–2/N0/M0 tumours ($P = 0.0007$). Cyclin A was also a powerful predictor of disease-free survival in T1–4/N0/M0 ($P = 0.0027$) tumours ($P = 0.0007$). Cyclin D1 and p21$^{(waf1/cip1)}$ were not significantly related to survival or disease-free survival in any of the groups. In the entire material the independent prognostic factors were the presence of distant metastases (relative risk (RR) 5.16, $P < 0.001$), T category (RR 2.68, $P < 0.001$), Ki-67 expression (RR 1.02, $P = 0.026$) and cyclin A expression (RR 1.12, $P = 0.001$). The independent predictors in T1–4/N0/M0 tumours were T-category (RR 2.67, $P = 0.001$) and cyclin A (RR 1.21, $P < 0.001$), and in T1–2/N0/M0 tumours the only significant predictor was cyclin A (RR 1.19, $P = 0.0002$). In renal cell carcinoma, cyclin A is a powerful and independent prognostic factor in all clinical stages of the disease, whereas cyclin D1 and p21$^{(waf1/cip1)}$ have no prognostic value.

**Keywords**: cyclin A; cyclin D1; p21 $^{(waf1/cip1)}$; cell proliferation; renal cell carcinoma

The main therapy in renal cell carcinoma (RCC) is operative treatment but the operative results are variable (Sweeney et al, 1996; Giberti et al, 1997). In a proportion of the cases, adjuvant treatment may be helpful to improve the survival of patients. To identify the group of patients who may benefit from additional treatment modalities, new accurate prognostic factors are urgently needed. Today the TNM classification and nuclear grade are the main factors on which the treatment decisions are based, but better prognostic factors are under research to tailor the therapy individually. The knowledge and understanding of the biological nature of RCC has increased and new promising prognostic indicators have been already identified. These markers include indicators of cell proliferation (Aaltomaa et al, 1997; Papadopoulos et al, 1997), cell adhesion (Koga et al, 1997; Paul et al, 1998), markers involved directly in the regulation of cell growth (Hofmockel et al, 1997; Shiina et al, 1997) and growth-stimulating or suppressing gene products (Hofmockel et al, 1997; Shiina et al, 1997).

The suppressor gene p53 is frequently mutated in different cancers and it is able to stop the cell cycle at G1 phase and also to induce apoptosis. The recent reports suggest that p21$^{(waf1/cip1)}$ is a main mediator of p53 tumour suppressor gene effects (EI Deiry et al, 1995; Naka et al, 1998). Elevated levels of p21$^{(waf1/cip1)}$ protein in response to both p53-dependent and -independent signals mediate the cell cycle arrest predominantly at the G1 phase of the cell cycle (Zeng et al 1996). p21$^{(waf1/cip1)}$ exerts this effect by inhibiting the cyclin-dependent kinases (cdk) that are required to drive the cell division cycle. While active cdk are found in normal cells in a quaternary complex with cyclins, proliferating cell nuclear antigen and p21$^{(waf1/cip1)}$, more than one molecule of p21$^{(waf1/cip1)}$ per complex results in kinase inhibition (Waga et al, 1994; Zhang et al, 1994). In experimental analyses of human bladder cancer cell lines p21$^{(waf1/cip1)}$ is able to induce apoptosis, but the exact role of p21$^{(waf1/cip1)}$ in apoptosis is not completely understood (Kawasaki et al, 1998). In RCC the significance of p21$^{(waf1/cip1)}$ protein in tumour differentiation and patient survival has not been previously studied. Cyclin A and D1 are protein kinases related to control of the cell cycle and cell proliferation (Donnellan and Chetty, 1998). Cyclins act together with the cdk by phosphorylating the main substrates responsible for the cell cycle regulation (Donnellan and Chetty, 1998). Phosphorylation of the retinoblastoma (Rb) tumour suppressor protein (pRB) by the complexes of cyclins and cdk makes Rb protein active to induce genes controlling cell proliferation, thus being a part of the control of the cell cycle machinery (Strauss et al, 1995; Donnellan and Chetty, 1998). Cyclin D1 is most active in G1 resting phase before S-phase by regulating the DNA synthesis, by phosphorylating the pRB and simultaneously activating its function (Strauss et al, 1995; Donnellan and Chetty, 1998). Cyclin A regulates the cell

*Received 11 November 1998*
*Revised 9 February 1999*
*Accepted 16 February 1999*

*Correspondence to:* S Aaltomaa cycle also through the pRB activation (Donnellan and Chetty, 1998). The overexpression of cyclins has been related to high proliferation rate of the tumour cells and to other unfavourable prognostic factors, although the results are not uniform (Aaltomaa et al, 1999*a*; Donnellan and Chetty, 1998; Ishikawa et al, 1998; Maeda et al, 1998; Volm et al, 1998). The gene amplification and protein expressions of cyclins A and D1 have been previously studied in several human epithelial neoplasms to establish their possible prognostic significance in clinical human cancer (Barbareschi et al, 1997; Michalides et al, 1997; Aaltomaa et al, 1999*a*; Donnellan and Chetty, 1998; Maeda et al, 1998; Volm et al, 1998). At present there are no reports available on the prognostic value of the expressions of cyclins A and D1 in RCC.

The aims of our study were to analyse the expressions of cyclins A and D1 as well as p21 $^{(waf1/cip1)}$ protein in RCC and to compare the expressions of these proteins to other clinical and histopathological parameters, and to establish the prognostic value of these proteins in RCC patients.

### PATIENTS AND METHODS

The study includes 118 RCC patients treated between 1968 and 1985 and followed up to 1991 at the Department of Surgery, Kuopio University Hospital, Finland. The cohort was not entirely consecutive since adequate biopsy specimens for immunohistochemistry were not available in all cases. The estimated total number of diagnosed RCCs during the enrolment period was about 200 cases, but an operation was carried out in only about half of the cases. The mean (range) age of the patients at diagnosis was 61 (range 22–82) years and the mean (range) follow-up time was 9.7 (range 5.3–20.2) years. The follow-up of patients after therapy lasted until death or to the year 1991. The diagnosis, clinical staging, treatment and clinical follow-up were carried out mainly by two urologists. The diagnosis was based on the results of routine laboratory tests and intravenous (i.v.) urography, and on ultrasonography and computerized tomography (CT) during the last years of follow-up. The histological confirmation was needed before the diagnosis was settled. In addition, the bone chart, chest X-ray and ultrasonography were done to detect the possible metastasis. During the first 2 years the follow-up was done on every 3rd month and thereafter every 6th month up to 5 years, and after that controls were started once a year. During the follow-up, routine laboratory tests and chest X-rays were done on regular basis, and ultrasonography, i.v. urography, CT and the bone chart when they were indicated. There were 114 cases treated with radical nephrectomy, in four cases a partial nephrectomy was done. The causes of death were verified from the patient files, death certificates and from the files of the Finnish Cancer Registry.

### Histological methods

Five micrometer-thick serial sections were cut from the paraffin-embedded surgical specimens and stained with haematoxylin and eosin. The samples were examined in a blinded manner. Tumours were categorized into four nuclear grades as described in detail in the previous literature (Syrjänen and Hjelt, 1978). The mitotic figures were identified, usually at the tumour periphery at the areas of the most proliferative and invasive growth. The counting of mitotic figures was done by using an objective magnification of 40 × (field diameter 490 μm) and the volume corrected mitotic index (M/V) method was used (Haapasalo et al, 1989). The M/V index expresses the number of mitotic figures $mm^{-2}$ of neoplastic epithelium in the section. The mean M/V index was 6.9 (standard deviation (s.d.) range 0–44) $mm^{-2}$.

### Cyclin A and D1 and p21($^{waf1/cip1}$) immunohistochemistry

Cyclin A (Aaltomaa et al, 1998a) was demonstrated by a routine immunohistochemical method, which is similar to that described in connection with Ki-67 immunostaining except that microwave pretreatment was not used in cyclin A immunohistochemistry. The antibody was purchased from Novocastra Laboratories (Newcastle upon Tyne, UK) and it was used at a dilution of 1:200. Cyclin D1 (Aaltomaa et al, 1999*a*) and p21$^{(waf1/cip1)}$ (Lipponen et al, 1998) were demonstrated by using the same staining procedure as described in connection with Ki-67 immunohistochemistry (Aaltomaa et al, 1997). Both of the antibodies were purchased from Novocastra Laboratories (Newcastle upon Tyne, UK) and they were used at a dilution of 1:100 (cyclin D1) and 1:20 (p21$^{(waf1/cip1)}$). The expressions of cyclins and p21$^{(waf1/cip1)}$ (fraction of positive tumour cells) were evaluated in the entire section (magnification 400 ×). The expression of cyclin D1 was considered positive only when distinct strong nuclear positivity was present. Faint expression of cyclin D1 was present in most of the cancers, but it was not included in the scoring process as recommended in the previous literature (Gillet et al, 1996). The mean (s.d.) fractions of cyclin A- and D1-positive nuclei were 2.2% (s.d. 4.1%) and 23.2% (s.d. 28.7%) respectively. The mean (s.d.) fraction of positive nuclei for p21$^{(waf1/cip1)}$ was 6.8% (s.d. 10.5%).

### Ki-67 immunohistochemistry

For immunohistochemical demonstration of Ki-67 protein, 5-μm sections were deparaffinized, rehydrated and washed for 5 min with phosphate-buffered saline (PBS). Thereafter the sections were rinsed in distilled water and heated in a microwave oven for 2 × 5 min in 0.01 M citrate buffer (pH 6.0). After that the slides were rinsed in Tris-buffered saline (pH 7.4). Endogenous peroxide was blocked by 3% hydrogen peroxide for 5 min followed by a wash for 5 min with PBS. The tissue sections were incubated with the monoclonal anti-Ki-67 protein (MIB1, Dianova Marseille, France) antibody diluted at 1:100 in PBS. Sections were washed twice for 5 min with PBS, incubated for 20 min with biotinylated secondary antibody (Vectastain ABC Elite Kit, Vector Laboratories, CA, USA) diluted at 1:200 in PBS. Slides were washed twice in PBS for 10 min and incubated for 20 min in preformed avidin–biotinylated peroxidase complex (Vectastain ABC Elite Kit, Vector Laboratories, CA, USA). Sections were washed twice for 5 min with PBS, developed with diaminobenzidine tetrahydrochloride substrate (Sigma Chemical Co., St Louis, MO, USA), slightly counterstained with Mayer's haematoxylin, dehydrated, cleared and mounted. Normal human tonsil was used as a positive control. The fraction of positively stained nuclei was estimated in the area of the tumour that contained the highest fraction of Ki-67-positive cells. The mean (s.d.) fraction of Ki-67 positive nuclei was 14.2% (s.d. 15.7%).

### p53 and PCNA immunohistochemistry

The expression of these proteins was detected by a routine immunohistochemical method as detailed in previous literature (Lipponen et al, 1994). The antibody for p53 (CM1, Novocastra

© 1999 Cancer Research Campaign

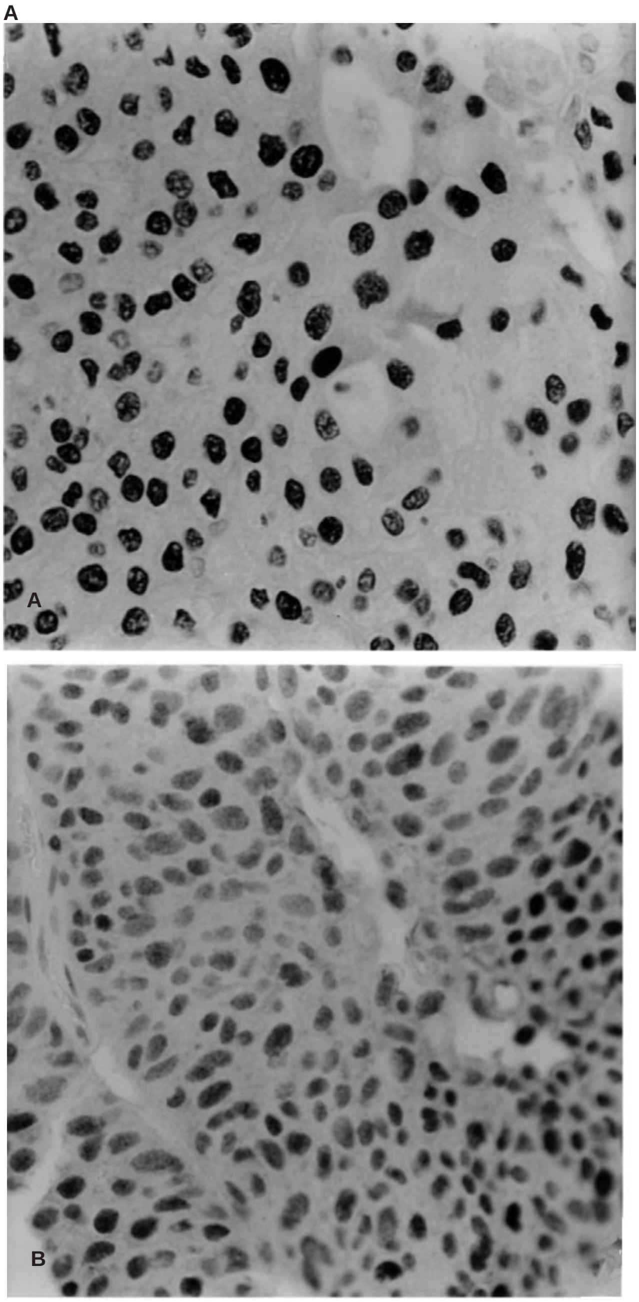

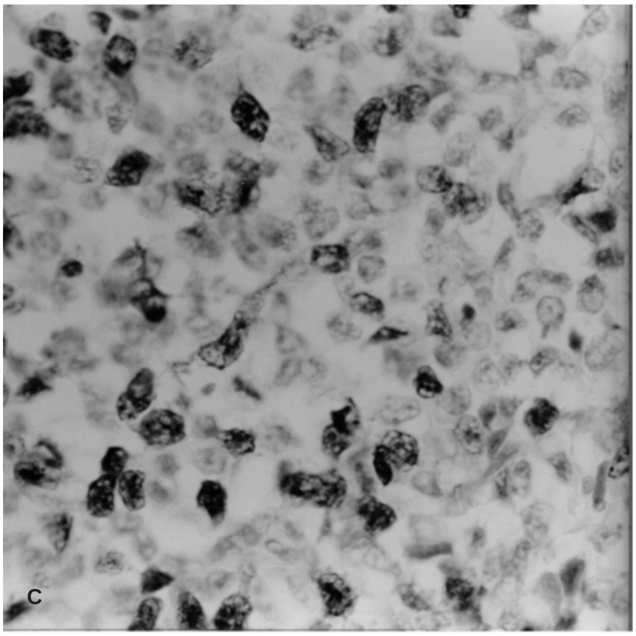

**Figure 1 (A)** The staining of cyclin D1 was always nuclear. A high fraction of cyclin D1-positive tumour cell nuclei in a poorly differentiated RCC (magnification 250×). (**B**)The staining of $p21^{(waf1/cip1)}$ protein was nuclear. A high fraction of positively stained tumour cell nuclei in RCC (magnification 250 ×). (**C**)The expression of cyclin A was both cytoplasmic and nuclear. A high fraction of cyclin A-positive tumour cell nuclei in a poorly differentiated RCC (magnification 250 ×)

Laboratories, Newcastle upon Tyne, UK) was used at a dilution of 1:1200. The antibody for PCNA (PC10) was purchased from DAKO (Golstrup, Denmark) and it was used at a dilution of 1:100. The fraction of positive cells was analysed from the areas with the highest fraction of positive cells as detailed before (Lipponen et al, 1994). The mean (s.d.) fraction of p53-positive cells was 1.2% (s.d. 7.6%) and of proliferating cell nuclear antigen (PCNA) 29.3% (s.d. 30.4%).

### Statistical methods

In statistical calculations, the SPSS-X was used in an IBM computer and the statistical tests used are indicated in the results when appropriate. Univariate survival analysis was done by the SPSS-X using the Kaplan–Meier method with statistics by Gehan. Multivariate survival analysis (Cox's analysis) was done in a stepwise manner. Survival analysis included (as an event) only the deaths due to renal cell carcinoma. During the follow-up, 68 patients died of renal cell carcinoma and three died from other causes. When the disease-free survival was analysed, only M0 cases (n = 82) were included in the analysis. The group limits (when categorized data is used) for Ki-67, p53 and PCNA were set so that these parameters showed the highest possible correlation to cyclin A, D and $p21^{(waf1/cip1)}$ expression in Table II.

### RESULTS

Cyclins A or D1 and $p21^{(waf1/cip1)}$ were not expressed in normal renal tissue adjacent to tumours. The positive stainings of cyclin D1 (Figure 1A) and $p21^{(waf1/cip1)}$ (Figure 1B) were confined to the tumour cell nuclei, whereas cyclin A (Figure 1C) was also expressed in the cytoplasm of the cancer cells. The staining pattern of these proteins showed both intra- and intertumour variation. The mean (range) fractions of cyclin A-, cyclin D1- and $p21^{(waf1/cip1)}$-positive cells were 2.2% (range 0–20%), 23.3% (range 0–90%) and 6.8% (range 0–70%), respectively.

The expression of $p21^{(waf1/cip1)}$ was not related to nuclear grade ($P = 0.6$), T-category ($P = 0.7$), M-category ($P = 0.4$), tumour size ($P = 0.8$), patient age ($P = 0.4$) or venous invasion ($P = 0.3$).

The expression of cyclins A and D1 were not related to sex, T-category, M-category or tumour size ($P > 0.05$), but the expression of cyclin A was related to venous invasion ($P = 0.04$) and cyclin D to age over 65 years ($P = 0.03$). The relationship between the histopathological parameters and cyclin A and D1 is shown in Table 1. The expression of $p21^{(waf1/cip1)}$ protein was not related to

© 1999 Cancer Research Campaign

**Table 1** The relationship between the fraction of cyclin A- and D1-positive cells and other prognostic factors in renal cell carcinoma

| Variable | Number | Cyclin A-positive cells mean (s.d.) | *P*-value[a] | Cyclin D1-positive cells mean (s.d.) | *P*-value[a] |
|---|---|---|---|---|---|
| NG 1 | 30 | 0.8 (2.7) | | 33.4 (34.7) | |
| NG 2 | 54 | 1.5 (3.2) | | 22.4 (26.8) | |
| NG 3 | 34 | 4.7 (5.4) | 0.0002 | 15.4 (24.4) | 0.042 |
| M/V ≤ 7/mm² | 84 | 1.4 (3.5) | | 26.1 (29.6) | |
| M/V > 7/mm² | 34 | 4.3 (4.8) | 0.002 | 16.2 (25.5) | 0.09 |
| Ki 67 < 1% | 17 | 0.1 (0.1) | <0.001 | 24.4 (33.4) | |
| Ki 67 > 1% | 92 | 2.6 (4.2) | | 22.8 (27.9) | 0.8 |
| PCNA < 5% | 48 | 1.1 (3.1) | 0.006 | 27.3 (31.9) | |
| PCNA > 5% | 63 | 3.2 (4.8) | | 20.6 (27.2) | 0.2 |
| p53 < 1% | 101 | 2.4 (4.4) | 0.4 | 21.3 (28.0) | |
| p53 > 1% | 10 | 1.4 (2.1) | | 46.0 (35.2) | 0.01 |
| p21 < 1% | 43 | 1.7 (3.9) | 0.3 | 13.6 (24.4) | |
| p21 > 1% | 75 | 2.6 (4.3) | | 28.7 (29.7) | 0.005 |

NG, nuclear grade; M/V, volume corrected mitotic index; PCNA, proliferating cell nuclear antigen. [a]Two groups: *t*-test; three groups: analysis of variance.

**Table 2** The correlation coefficients and their significance between the different histopathological tumour markers.

| | M/V | KI-67 | PCNA | p53 | Cyclin A | Cyclin D1 |
|---|---|---|---|---|---|---|
| Cyclin A | 0.4574 | 0.6295 | 0.4304 | –0.0438 | | |
| | *P* = 0.235 | *P* = 0.000 | *P* = 0.000 | *P* = 0.648 | | |
| Cyclin D1 | –0.1359 | –0.1659 | –0.2078 | 0.0338 | –0.104 | |
| | *P* = 0.142 | *P* = 0.085 | *P* = 0.029 | *P* = 0.725 | *P* = 0.25 | |
| p21 | 0.1009 | 0.1668 | 0.1200 | –0.0693 | 0.0547 | 0.2334 |
| | *P* = 0.277 | *P* = 0.083 | *P* = 0.210 | *P* = 0.470 | *P* = 0.556 | *P* = 0.011 |

M/V index, mitotic index: PCNA, proliferating cell nuclear antigen.

**Table 3** The independent prognostic factors (final step analysis) of survival in multivariate analysis, which included all the analysed prognostic parameters. Some of the parameters were not available in all cases and accordingly the total number of cases included is lower than in the original cohort

| Variable | RR | CI | *P*-value |
|---|---|---|---|
| Entire cohort (*n* = 96) | | | |
| Metastasis | 5.16 | 2.71–9.83 | <0.001 |
| T-category | 2.68 | 1.67–4.31 | <0.001 |
| Ki-67 | 1.02 | 1.00–1.04 | 0.026 |
| Cyclin A | 1.12 | 1.05–1.20 | 0.001 |
| M0 tumours (*n* = 70) | | | |
| T-category | 2.67 | 1.48–4.84 | 0.001 |
| Cyclin A | 1.21 | 1.12–1.31 | <0.001 |

Multivariate analyses included following variables: T-classification, node status, presence of metastasis, nuclear grade, mitotic rate, Ki-67, cyclin A and D1, p21[(waf1/cip1)], p53, proliferating cell nuclear antigen (PCNA), sex and age. RR, risk ratio; CI, 95% confidence interval.

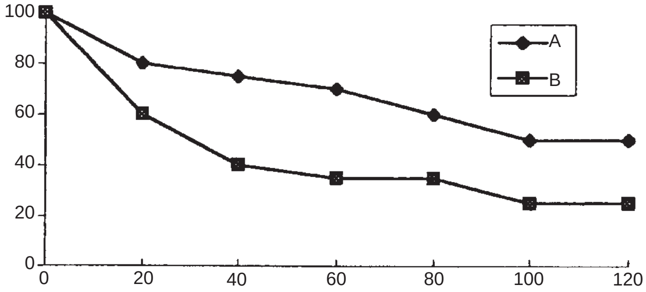

**Figure 2** The survival of RCC patients categorized according to the fraction of cyclin A-positive cancer cells in the entire study group. The curves are significantly separated ($\chi^2$ = 10.2, *P* = 0.0014). Curve A: the fraction of cyclin A-positive cells ≤ 1%, *n* = 66; Curve B: the fraction of cyclin A-positive cells > 1%, *n* = 47

any of the parameters shown in Table 1. The correlation coefficients and their significance between the different histopathological tumour markers are shown in Table 2.

Cyclin A predicted survival in the entire study group (*P* = 0.0014, Figure 2), in M0 (*P* = 0.0007, Figure 3) and in T1–2/N0/M0 cases (*P* = 0.0007, Figure 4). It was also a powerful predictor of disease-free survival in T1–4/N0/M0 (*P* = 0.0027) and in T1–2/N0/M0 tumours (*P* = 0.0007). Cyclin D1 and p21[(waf1/cip1)] were not significantly related to patient survival or disease-free survival in any of the analysed subgroups. The independent prognostic factors of survival are shown in Table 3.

### DISCUSSION

Cyclins are closely related to cell cycle by acting together with cdk in phosphorylating the Rb protein. Type D1 cyclin is mostly expressed during the first gap phase (G1) of the cell cycle and it is a major factor in the timing process of starting DNA synthesis in mammalian cells (Strauss et al, 1995; Kato, 1997; Donnellan and Chetty, 1998). On the contrary, cyclin A is expressed mainly in late S and G2 phases and is degraded during mitotic phase before

 © 1999 Cancer Research Campaign

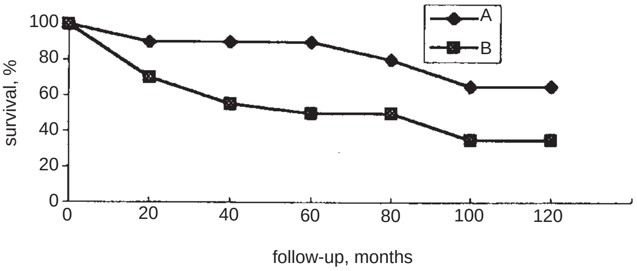

**Figure 3** The survival of RCC patients categorized according to the fraction of cyclin A-positive cancer cells in M0 patients. The curves are significantly separated ($\chi^2$ = 11.5, $P$ = 0.007). Curve A: the fraction of cyclin A-positive cells ≤ 1%, $n$ = 50; Curve B: the fraction of cyclin A-positive cells > 1%, $n$ = 32

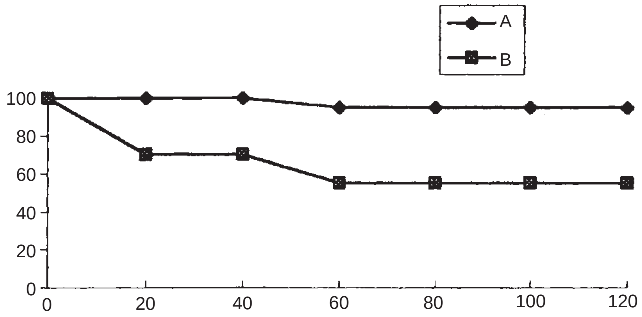

**Figure 4** The survival of patients with a T1–2/N0/M0 RCC categorized according to the fraction of cyclin A-positive cancer cells. The curves are significantly separated ($\chi^2$ = 11.4, $P$ = 0.0007). Curve A: the fraction of cyclin A-positive cells ≤1%, $n$ = 29; Curve B: the fraction of cyclin A-positive cells > 1%, $n$ = 16

the metaphase (Donnellan and Chetty, 1998). The increased expression of cyclins is related to disregulated or accelerated cell cycle as a result of gene amplification, chromosomal translocation, down- or up-regulated expression (Naitoh et al, 1995; Barbareschi et al, 1997; Aaltomaa et al, 1999*a*; Volm et al, 1998). So far, no previous study has compared the relationships between the expressions of cyclins and p21(waf1/cip1), p53 as well as Ki-67 expressions in RCC. Consequently, the prognostic role of cyclins in RCC has not yet been defined.

Although in other carcinomas the relationship between TNM classification and cyclins is obvious (Michalides et al, 1997; Aaltomaa et al, 1999*a*), we could not find any correlation between the expression of cyclin A and TNM categories. Differentiation grade was significantly associated with the fraction of cyclin A-positive cells, which is in line with the results from prostate cancer as well (Aaltomaa et al, 1999*a*). The expression of cyclin A was also related to many indicators of cell proliferation such as Ki-67 and PCNA expressions and mitotic rate, a finding which is in accordance with previous reports (Marshall et al, 1996; Aaltomaa et al, 1999*a*). The staining signal of cyclin A was independent of p53 and p21(waf1/cip1) expressions, which suggests that these latter proteins may be of minor importance in cell cycle control in RCC. Additional support for this suggestion comes from the study (Papandreou et al, 1997) in which the p21(waf1/cip1) gene was rarely mutated in RCC.

Cyclin D1 was not related to TNM classification in RCC in the present study. A high fraction of cyclin D1-positive cells was linked with low cell proliferation and well-differentiated tumours. There are no reports available in the literature to verify our results in RCC, but in breast cancer the expression of cyclin D1 is related to sex steroid receptor positivity and favourable histological signs (Gillet et al, 1996; Barbareschi et al, 1997). Also, in bladder cancer, the initial results suggest that cyclin D1 might be associated with low histological grade (Lee et al, 1997). The above results in bladder and breast cancer are in accordance with our results in RCC. However, the results are variable, since the close relationship between overexpression of cyclin D1 and malignant cellular prognostic features has been observed in other cancers (Nakamura et al, 1997; Pignataro et al, 1998). In gastric and colon cancer the strong expression of cyclin D1 is related to lymph node involvement (Nakamura et al, 1997; Maeda et al, 1998), and in ovarian cancer to tumour malignancy (Barbieri et al, 1997). In addition, our analysis revealed a positive correlation (Table 2) between the expression of p21(waf1/cip1) and cyclin D1. This association is most probably due to reduced cell proliferation of cyclin D1-positive tumours and possibly to growth inhibitory functions of p21(waf1/cip1).

In RCC, the staining signal of p21(waf1/cip1) seems to be independent of p53 expression. Indeed, p53 independent expression of p21(waf1/cip1) has been reported in several tumour types, including colon, endometrium, prostate and bladder cancer (Backe et al, 1997; Yasui et al, 1997; Aaltomaa et al, 1999*b*; Lipponen et al, 1998). Unexpectedly, p21(waf1/cip1) was not associated with the expression of PCNA or other indicators of accelerated cell proliferation, although in cells p21(waf1/cip1) exists as a protein complex together with PCNA, cyclin and cdk. The data relating p21(waf1/cip1) to cell proliferation are rather variable (Backe et al, 1997; Aaltomaa et al, 1999*b*; Lipponen et al, 1998). In bladder cancer, p21(waf1/cip1) was related to high cell proliferation, but in another study no significant relationship could be found between these variables (Lipponen et al, 1998). These conflicting results are most probably due to altered stoichiometry of these proteins in malignant cells, and the metabolism of p21(waf1/cip1) may be disturbed because of an altered protein structure. Also, it is possible that p21(waf1/cip1)-positive tumour cells represent a subpopulation of cells that have withdrawn from the cell cycle amid the otherwise rapidly proliferating tumour cells (El Deiry et al, 1995).

Our survival analysis showed that p21(waf1/cip1) had no prognostic value in RCC. Similar results have been reported in bladder, prostate, endometrium and hepatocellular cancer (Backe et al, 1997; Byrne et al, 1997; Aaltomaa et al, 1999*b*; Lipponen et al, 1998; Naka et al, 1998). On the contrary, in oesophageal and colon cancer, as well as in squamous cell carcinomas of the head and neck, the strong expression of cyclin D1 was related to unfavourable prognosis (Ishikawa et al, 1998; Maeda et al, 1998). In addition, in colorectal adenocarcinoma and in superficial bladder cancer the overexpression of cyclin D1 predicts early recurrence (Shin et al, 1997; Maeda et al, 1998). Moreover, cyclin D1 overexpression is related to favourable prognosis in breast cancer (Gillet et al, 1996). In RCC we found no correlation between the expression of cyclin D1 and survival or recurrence-free survival, whereas cyclin A was a powerful predictor of patient outcome, and the independent prognostic value of cyclin A was confirmed also in multivariate analyses of the various subgroups of the tumours. If the cases were categorized into three groups – 0%, 1–5% and over 5% – of cyclin A-positive cells, the corresponding survival rates at 10 years were 60%, 30% and 10% respectively ($P$ < 0.0001). This clearly shows that cyclin A expression can be used in categorizing RCCs into prognostic groups.

© 1999 Cancer Research Campaign

Cyclin A was an important prognostic factor for recurrence-free survival as well. Since there are no published reports available on the prognostic value of cyclin A in other cohorts of RCC, the comparison to our data remains to be verified in further studies. However, there are only few prognostic studies of abnormal expression of cyclin A in the literature and similar prognostic results have been reported at least in squamous cell lung and prostate cancer (Aaltomaa et al, 1999*a*; Volm et al, 1998).

Finally, we suggest that cyclin A expression is a valuable additional prognostic factor in all stages of RCC and might be used as an additional prognostic criteria in defining correct prognostic category for patients to be used in making therapy plans.

In conclusion, the expression of cyclin A was closely related to malignant cellular features and cell proliferation in RCC. The staining signal of cyclin D1 showed a weak inverse correlation to indicators of cell proliferation and tumour malignancy, while the expression of p21$^{(waf1/cip1)}$ was totally independent of other prognostic factors. Moreover, cyclin A overexpression was independently associated to shortened recurrence-free survival and cancer related survival, suggesting that cyclin A is a promising prognostic factor in renal cell carcinoma.

### ACKNOWLEDGEMENTS

The technical assistance of Ms Aija Parkkinen is greatly acknowledged. This study was financially supported by a research grant (EVO funding) from Kuopio University Hospital.

### REFERENCES

Aaltomaa S, Lipponen P, Vesalainen S, Eskelinen M and Syrjänen K (1997) Value of Ki-67 expression in renal cell carcinomas. *Eur Urol* **32**: 350–355

Aaltomaa S, Eskelinen M and Lipponen P (1999*a*) Expression of cyclin A and D proteins in prostate cancer and their relation to clinicopathological variables and patient survival. *Prostate* **30**: 175–182

Aaltomaa S, Lipponen P, Eskelinen M, Ala-Opas M and Kosma V-M (1999*b*) The prognostic value and expression of p21$^{(waf/cip1)}$ protein in prostate cancer. *Prostate* **39**: 8–15

Backe J, Gassel AM, Hauber K, Krebs S, Bartek J, Caffier H, Kreibe HH, Muller-Hermelink HK and Dietl J (1997) p53 protein in endometrial cancer is related to proliferative activity and prognosis but not to expression of p21 protein. *Int J Gynecol Pathol* **16**: 361–368

Barbareschi M, Pelosio P, Caffo O, Buttitta F, Pellegrini S, Barbazza R, Dalla Parma P, Bevilacqua G and Marchetti A (1997) Cyclin D1-gene amplification and expression in breast carcinoma: relation with clinicopathologic characteristics and with retinoblastoma gene product, p53 and p21/waf1 immunohistochemical expression. *Int J Cancer* **74**: 171–174

Barbieri F, Cagnoli M, Ragni N, Pedulla F, Foglia G and Alama A (1997) Expression of cyclin D1 correlates with malignancy in human ovarian tumors. *Br J Cancer* **75**: 1263–1268

Byrne RL, Horne CH, Robinson MC, Autzen P, Apakama I, Bishop RI, Neal DE and Hamdy FC (1997) The expression of waf-1, p53 and bcl-2 in prostatic adenocarcinoma. *Br J Urol* **79**: 190–195

Donnellan R and Chetty R (1998) Cyclin D1 and human neoplasia. *J Clin Pathol Mol Pathol* **51**: 1–7

El Deiry WS, Tokino T and Waldman T (1995) Topological control of p21$^{(waf1/cip1)}$ expression in normal and neoplastic tissues. *Cancer Res* **55**: 2910–2919

Giberti C, Oneto F, Martorana G, Rovida S and Carmignani G (1997) Radical nephrectomy for renal cell carcinoma: long-term results and prognostic factors on a series of 328 cases. *Eur Urol* **31**: 40–48

Gillet CE, Smith P, Gregory WM, Richards MA, Millis RR, Peters G and Barnes DM (1996) Cyclin D1 and prognosis in breast cancer. *Int J Cancer* **69**: 92–99

Haapasalo H, Pesonen E and Collan Y (1989) Volume corrected mitotic index (M/V-INDEX): the standard of mitotic activity in neoplasms. *Path Res Prac* **185**: 551–554

Hofmockel G, Bassukas ID, Wittmann A and Dämmrich J (1997) Is the expression of multidrug resistance gene product a prognostic indicator for the clinical outcome of patients with renal cell cancer? *Br J Urol* **80**: 11–17

Ishikawa T, Furihata M, Ohtsuki Y, Murakami H, Inoue A and Ogoshi S (1998) Cyclin D1 overexpression related to retinoblastoma protein expression as a prognostic marker in human oesophageal squamous cell carcinoma. *Br J Cancer* **77**: 92–97

Kato JY (1997) Control of G1 progression by D-types cyclins: key event for cell proliferation. *Leukemia* **3**: 347–351

Kawasaki T, Tomita Y, Bilim V, Takeda M, Takahashi K and Kumanishi T (1998) Abrogation of apoptosis induced by DNA-damaging agents in human bladder-cancer cell lines with p21/waf1/cip1 and/or p53 gene alterations. *Int J Cancer* **68**: 501–505

Koga H, Naito S, Hasegawa S, Watanabe T and Kumazawa J (1997) A flow cytometric analysis of the expression of adhesion molecules on human renal cell carcinoma cells with different metastatic potentials. *Eur Urol* **31**: 86–91

Lee CC, Yamamoto S, Morimura K, Wanibuchi H, Nishisaka N, Ikemoto S, Nakatani T, Wada S, Kishimoto T and Fukushima S (1997) Significance of cyclin D1 overexpression in transitional cell carcinomas of the urinary bladder and its correlation with histopathologic features. *Cancer* **15**: 780–789

Lipponen P, Aaltomaa S, Eskelinen M, Ala-Opas M and Kosma V-M (1998) Expression of p21 $^{(waf1/cip1)}$ protein in transitional cell bladder tumours and its prognostic value. *Eur Urol* **34**: 237–243

Lipponen P, Eskelinen M, Hietala K and Syrjänen K (1994) Expression of proliferating cell nuclear antigen (PC10), p53 protein and c-erbB-2 in renal adenocarcinoma. *Int J Cancer* **57**: 275–280

Maeda K, Chung Y, Kang S, Ogawa M, Nishiguchi Y, Ikehara T, Nakata B, Okuno M and Sowa M (1998) Cyclin D1 overexpression and prognosis in colorectal adenocarcinoma. *Oncology* **55**: 145–151

Marshall RD, Lester S, Corless C, Richie JP, Chandra R, Propert KJ and Dutta A (1996) Expression of cell cycle-regulated proteins in prostate cancer. *Cancer Res* **56**: 4159–4163

Michalides RJ, van Veelen NM, Kristel PM, Hart AA, Loftus BM, Hilgers FJ and Balm JA (1997) Over-expression of cyclin D1 indicates poor prognosis in squamous cell carcinoma of the head and neck. *Arch Otolaryngol Head Neck Surg* **123**: 497–502

Naitoh H, Shibata J, Kawaguchi A, Kodama M and Hattori T (1995) Overexpression and localization of cyclin D1 mRNA and antigen in esophageal cancer. *Am J Pathol* **146**: 1161–1169

Naka T, Toyota N, Kaneko T and Kaibara N (1998) Protein expression of p53, p21WAF1, and Rb as prognostic indicators in patients with surgically treated hepatocellular carcinoma. *Anticancer Res* **18**: 555–564

Nakamura M, Katano M, Fujimoto K and Morisaki T (1997) A new prognostic strategy for gastric carcinoma: mRNA expression of tumor growth-related factors in endoscopic biopsy specimens. *Ann Surg* **226**: 35–42

Papandopoulos I, Rudolph P and Weichert-Jacobsen K (1997) Value of p53 expression, cellular proliferation and DNA content as prognostic indicators in renal cell carcinoma. *Eur Urol* **32**: 110–117

Papandreou CN, Bogenrieder T, Loganzo F, Albino AP and Nanus DM (1997) Expression and sequence analysis of the p21 $^{(waf1/cip1)}$ gene in renal cancers. *Urology* **49**: 481–486

Paul R, Ewing CM, Robinson JC, Marshall FF, Johnson KR, Wheelock MJ and lsaacs WB (1998) Catherin-6, a cell adhesion molecule specifically expressed in the proximal renal tubule and renal cell carcinoma. *Cancer Res* **57**: 2741–2748

Pignataro L, Pruneri G, Carboni N, Capaccio P, Cesana BM, Neri A and Butler MR (1998) Clinical relevance of cyclin D1 protein expression in laryngeal squamous cell carcinomas. *J Clin Oncol* **16**: 3069–3077

Shiina H, Igawa M, Urakami S, Ishibe T and Kawanishi M (1997) Clinical significance of immunohistochemically detectable p53 protein in renal cell carcinoma. *Eur Urol* **31**: 73–80

Shin KY, Kong G, Kim WS, Lee TY, Woo YN and Lee JD (1997) Over-expression of cyclin D1 correlates with early recurrence in superficial bladder cancer. *Br J Cancer* **75**: 1788–1792

Strauss M, Lukas J and Bartek J (1995) Unrestricted cell cycling and cancer. Recent data suggest that de-regulation of the restriction point in the cell cycle G1 phase appears to be required for the development of neoplasia. *Nat Med* **1**: 1245–1246

Sweeney JP, Thornhill JA, Grainger R, Mcdermott TED and Butler MR (1996) Incidentally detected renal cell carcinoma: pathological features, survival trends and implications for treatment. *Br J Urol* **78**: 351–353

Syrjänen K and Hjelt L (1978) Grading of human renal adenocarcinoma. *Scand J Urol Nephrol* **12**: 49–52

Volm M, Koomagi R and Rittgen W (1998) Clinical implication of cyclins, cyclin-dependent kinases, RB and E2F1 in squamous-cell lung carcinoma. *Int J Cancer* **79**: 294–299

 *© 1999 Cancer Research Campaign*

Waga S, Hannon GJ, Beach D and Stillman B (1994) The p21 inhibitor of cyclin dependent kinases controls DNA replication by interaction with PCNA. *Nature* **369**: 574–578

Yasui W, Akama Y, Yokozaki H, Semba S, Kudo Y, Shimamoto F and Tahara E (1997) Expression of p21 waf1/cip1 in colorectal adenomas and adenocarcinomas and its correlation with p53 protein expression stage. *Pathol Int* **47**: 470–477

Zhang H, Hannon GJ and Beach D (1994) p21-containing cyclin kinases exist in both active and inactive states. *Genes Dev* **8**: 1750–1758

Zeng Y-X and El-Deiry WS (1996) Regulation of p21 $^{(waf1/cip1)}$ expression by p53-independent pathways. *Oncogene* **12**: 1557–1564

*© 1999 Cancer Research Campaign*
